# Chronic periadolescent leuprolide exposure affects the development of reproductive physiology and behavior of female and male rats differently, but both mature after treatment termination

**DOI:** 10.1186/s13293-022-00485-5

**Published:** 2023-01-06

**Authors:** Fay A. Guarraci, Layla Avendano, Megan Kelly, Cleriza Estoesta, Bernard Sencherey, Hannah S. Valdivia, Amanda Gale, Lily Yepez, Jasmine B. Belfield, Kristen M. Carter, Natalie Williams, Andrea C. Gore

**Affiliations:** 1 Department of Psychology, Southwestern University, Georgetown, TX 78626 USA; 2 Department of Biology, Francis Marion University, Florence, SC 29506 USA; 3 Division of Pharmacology and Toxicology, The University of Texas, at Austin, Austin, TX 78712 USA

**Keywords:** GnRH agonist, Partner preference, Sexual motivation, Estrous cycle, Vaginal opening, Preputial separation, Puberty, Paced mating, Copulatory behavior, Sexual behavior

## Abstract

**Background:**

GnRH agonists have been used to halt the development of puberty in children with precocious puberty since the 1980s. Recently, drugs like Lupron Depot^®^ (leuprolide acetate), have been used to suppress pubertal progression in adolescents who are questioning their gender identity. However, few preclinical studies have been conducted to investigate potential effects of using GnRH agonists in this context.

**Methods:**

The present study tested the effects of daily leuprolide treatment (50 µg/kg, postnatal day (PD) 25–50) on pubertal onset in female (i.e., vaginal opening) and male (i.e., preputial separation) Long-Evans rats. The first estrous cycle immediately after vaginal opening was also measured. Sexual behavior and sexual motivation were tested using the partner-preference paradigm. Female rats were tested during the first behavioral estrus after treatment ended (between PD 51–64). Male rats were tested weekly for four consecutive weeks starting three days after treatment ended (PD 53).

**Results:**

Consistent with previous findings, leuprolide significantly delayed pubertal onset in both female and male rats. In addition, the first estrous cycle during the treatment period was disrupted by leuprolide, as indicated by a failure to cycle into estrus after vaginal opening until treatment ended. However, leuprolide affected neither sexual motivation nor fertility when female rats were tested within 14 days of leuprolide treatment ending. In contrast, the development of copulatory behavior and sexual motivation was significantly delayed by leuprolide in male rats; however, mature reproductive behavior was observed by the fourth week post-treatment.

**Conclusions:**

Taken together with previous findings, the present results indicate that male rats may be more sensitive to periadolescent leuprolide administration, taking longer to overcome the effects of leuprolide than female rats. Nevertheless, not long after leuprolide treatment is discontinued, sex-typical reproductive physiology and behavior emerge fully in female and male rats, indicating that the drug’s effects are not permanent. If translatable to humans, leuprolide may be a reversible option to give adolescents more time to consider their gender identity with minimal long-term effects on sexual development.

## Highlights

- Leuprolide acetate (50 µg/kg, postnatal day 25–50) suppresses pubertal development in female and male rats.
- Estrous cyclicity is suppressed during treatment in female rats.
- Development of male copulatory behavior and sexual motivation is significantly delayed but reaches control levels within a month of treatment termination.
- Female sexual motivation and fertility are unaffected by leuprolide once treatment ends.

## Background

Gonadotropin-releasing hormone (GnRH) agonists, such as leuprolide acetate (known by the brand name Lupron Depot^®^), are prescribed to children diagnosed with central precocious puberty to suppress continued pubertal development [1, 2]. More recently, GnRH agonists have been prescribed to adolescents who do not identify with the gender they were assigned at birth [3, 4]. Blocking the progression of puberty through the Tanner stages temporarily delays the emergence of secondary sex characteristics [3, 5]. Delaying physical manifestations of puberty reduces distress [3], improves well-being [6], and provides a diagnostic period for adolescents questioning their gender identity until they are ready to consider steps for more permanent gender transition in adulthood (e.g., gender-affirming hormones, surgery) [6]. If adolescents take leuprolide during this diagnostic period, they can more easily live in their experienced gender [6], while preventing the development of unwanted permanent physical changes [4, 7]. GnRH agonists can also be used for long-term suppression of endogenous gonadal hormone secretion once gender-affirming hormones are prescribed [8].

Only a few preclinical studies have tested the effects of blocking puberty in animals to model the use of GnRH agonists to halt pubertal progression in adolescents questioning their gender identity. For instance, in male rats, daily leuprolide treatment during the periadolescent period delayed puberty, as well as the development of copulatory behavior and sexual motivation when preference for a sexual partner was tested within 3 days of treatment termination [9]. Similarly, male mice exposed to leuprolide for 6 weeks in late adolescence spent less time investigating a female stimulus, but more time investigating a male stimulus compared to saline controls [10]. These results suggest that leuprolide delayed puberty, as well as the maturation of sexual behavior and sexual motivation in male rodents.

In contrast, leuprolide seems to have no effect on sociosexual behavior in female rodents. For example, we failed to find any long-term effects of daily periadolescent leuprolide administration on sexual behavior or sexual motivation when preference for a sexual partner was tested in female rats two months after leuprolide treatment ended [11]. Nor did we find any lasting effects of leuprolide on estrous cyclicity when measured for a month, starting two weeks after leuprolide exposure ended [11]. Similarly, Anacker et al. [10] found no effect of leuprolide on social preference in female mice. Specifically, both groups of female mice (leuprolide-treated and saline controls) spent equal amounts of time investigating a male and a female stimulus when tested during 6 weeks of leuprolide treatment in late adolescence. However, estrous cyclicity was halted during leuprolide exposure. Taken together, these results indicate that pubertal suppression might have more robust effects on the maturation of copulation and sexual motivation in males than in females. Nevertheless, differences in the timing of testing relative to the timing of leuprolide exposure may explain these sex and species differences. Furthermore, it is still unclear from this limited number of studies when reproductive functioning fully emerges after leuprolide treatment ends.

Therefore, the present study was designed to investigate the effects of daily leuprolide treatment on pubertal onset, sexual behavior and sexual motivation in head-to-head studies in female and male rats, using the same dose and exposure period (50 µg/kg; PD 25–50). In female rats, we assessed estrous cyclicity during leuprolide treatment, preference for a sexual partner after leuprolide treatment ended (PD 51–64), as well as pregnancy and parturition outcomes. In male rats, we measured sexual behavior and sexual motivation for 4 weeks to investigate the potential for complete maturation of reproductive behavior in the present study.

## Methods

### Animals

Female rats (*n* = 24) and male rats (*n* = 16) were bred from adult Long-Evans rats purchased from Envigo (Indianapolis, IN, USA). All experimental procedures were approved by the Southwestern University IACUC and followed NIH guidelines. Additional adult female and male rats were purchased from Envigo and used as stimulus rats. Stimulus rats were gonadally intact, sexually experienced male and female rats, with female stimulus rats only being used when in behavioral estrus (i.e., estrus phase of the estrous cycle). All rats were group-housed with same-sex cage mates in hanging polycarbonate cages. Aspen wood shavings were used for bedding and a red plastic tube was placed in each cage for enrichment. Food and water were available ad libitum. Temperature and humidity in the colony were controlled and monitored daily. The lights in the colony were maintained on a reversed 12:12 h light–dark cycle (with lights off at 10:00 a.m.). Mating tests occurred during the dark phase of the light–dark cycle, under dim red light.

### Experimental design

**Fig. 1:**
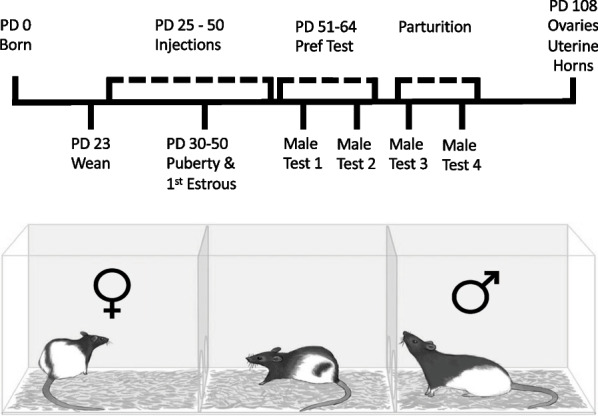
Timeline of procedures. Top: timeline starts at birth of rats in the laboratory and includes leuprolide treatment, puberty determination, and monitoring estrous cyclicity. Time frame for testing partner preference is noted as well as when organs were removed. (PD = postnatal day). Bottom: diagram of the apparatus used to measure partner preference. The experimental subject is located in the center compartment and the stimulus rats are depicted in the side compartments

Female pups and male pups from 6 l were randomly assigned to receive either leuprolide acetate (50 µg/kg dissolved in 0.9% sterile physiological saline; Sigma-Aldrich Corporation, St. Louis, MO, USA; female pups: *n* = 10; male pups: *n* = 8) or sterile physiological saline (1 ml/kg 0.9% NaCl; female pups: *n* = 14; male pups: *n* = 8) starting on PD 25 [12]. Subjects received subcutaneous injections daily at 9:00 a.m. between PD 25–PD 50. Although adolescent humans questioning their gender identity typically start taking puberty blockers after the initial signs of puberty have emerged (i.e., Tanner stage 2) [6], we administered leuprolide just prior to the physical signs of puberty in female and male rats because there are few easily identifiable indicators of pubertal onset in rats. Additionally, we wanted to confirm the effectiveness of leuprolide on pubertal delay. Starting on PD 30, signs of puberty were observed daily (i.e., vaginal opening for female subjects and preputial separation for male subjects). See Fig. 1 top for a timeline of experimental procedures.

For female subjects, the first estrous cycle was measured starting the day after vaginal opening. Female subjects were tested for partner preference on their first day in behavioral estrus after treatment termination with screening starting on PD 51. We chose to study rats in behavioral estrus to evaluate the effects of leuprolide on natural fluctuations in ovarian hormones. We restricted behavioral assessment to a two-week period following the last day of treatment (PD 51–64) to best assess the first behavioral estrus following treatment termination. Pregnancy and parturition were monitored during the 22 days following mating behavior. To assess any effects of leuprolide on ovarian and uterine horn weights, all female subjects were  anethestized and ovaries and uterine horns were removed and weighed on PD 108. See Fig. 1 top. For male subjects, the development of sexual behavior and sexual motivation was assessed weekly for 4 consecutive weeks, starting on PD 53 using the partner-preference paradigm.

### Puberty determination

Female subjects were examined daily for signs of vaginal opening, which is an external sign of the first estrous cycle [13] and typically expected anytime between PD 30 and PD 42 [14]. Male subjects were examined daily for signs of preputial separation (PPS), which is the ability to retract the prepuce by applying gentle pressure from the thumb and index finger [15], as an external sign of pubertal onset. Preputial separation is typically expected anytime between PD 39 and PD 44 [15]. All observations were conducted by trained observers who were blind to each subject’s drug treatment condition.

### Estrous cyclicity

For female subjects, the first estrous cycle was assessed from daily observations of vaginal epithelial cells starting the day after vaginal opening. The tip of a transfer pipette was filled with saline and placed into the vagina to collect epithelial cells. The vagina was flushed gently with saline [16]. This vaginal fluid was then collected in the pipette tip and placed onto a glass slide for examination under a brightfield microscope. Stages of the estrous cycle were determined by observing cell types in each sample of vaginal fluid. Proestrus is defined by the presence of predominantly nucleated epithelial cells, which can appear in clusters or individually [16]. Estrus is defined by the presence of predominantly cornified squamous epithelial cells in clusters [16]. Metestrus is defined by the presence of a mix of cells including leukocytes, nucleated epithelial, and cornified squamous epithelial cells [16]. Diestrus is defined by the presence of predominantly leukocytes [16]. Multiple observers, blind to drug treatment, assessed every sample for every subject for inter-rater reliability.

### Acclimation

All subjects were acclimated to the mating chambers twice, starting one week prior to partner-preference tests. Each mating chamber was made from a Plexiglas arena (91 cm long × 31 cm high × 37 cm wide) that was divided into three equally sized compartments using two clear Plexiglas dividers. Each of these clear Plexiglas dividers (30 cm high by 37 cm wide) had two 5-cm holes, one in each bottom corner. See Fig. 1 bottom. Aspen wood shavings covered the floor. During each acclimation session, one subject was placed into the center compartment and allowed to move freely between the three compartments of the mating chamber for 15 min.

Stimulus rats were trained to not exit through the holes in the clear Plexiglas dividers prior to use in any mating tests. During each of these training sessions, a sexually experienced gonadally intact female stimulus was placed in one of the side compartments and a sexually experienced gonadally intact male stimulus was placed in the other side compartment for 15 min. The stimulus rats were tapped lightly on the nose if they attempted to exit through the holes in the clear Plexiglas dividers. Four training sessions are typically required to teach the stimulus rats to remain in their compartment. If any stimulus attempted to exit through the holes during a mating test, a reminder tap was applied.

### Partner-preference test and paced-mating behavior

For all tests, subjects were given a choice between spending time with a same-sex or an opposite-sex stimulus, from which partner preference was determined. In female subjects, testing happened on the first day of behavioral estrus during the two-week period following the end of treatment (between PD 51 and PD 64). Because female subjects were allowed to mate with the male stimulus during this test, we were able to measure paced-mating behavior, whereby the female subject could pace the receipt of sexual stimulation (mounts, intromissions, ejaculations) or interact with a female stimulus. Female subjects could only be tested once because paced mating led to pregnancy in many subjects. Male subjects were tested once per week for a total of four tests (on PD 53, PD 60, PD 67, and PD 74) to assess the development of copulatory behavior and sexual motivation, which typically requires multiple mating tests to fully emerge. Because male subjects were allowed to mate with the female stimulus during these tests for partner preference, we were able to measure copulatory behaviors, as well as interactions with a male stimulus.

Immediately prior to each test, stimulus animals were confirmed to be sexual vigorous (i.e., female stimulus animals were sexually receptive; male stimulus animals mounted) and only used once per test day. Male subjects were tested with different, unfamiliar stimulus animals for each of the four tests.

For each partner-preference test, a subject was placed into the center compartment of the mating chamber and initially confined there with two opaque Plexiglas dividers. The opaque Plexiglas dividers covered the holes in the clear Plexiglas dividers, preventing the subject from entering either of the two side compartments, each of which held a female or male stimulus. The location of each stimulus was randomly assigned. The test started when the two opaque dividers were removed, thus allowing the subject full physical access to either the female or male stimulus and the ability to control the rate of sexual behaviors. After 10 min, the opaque dividers were replaced and all of the rats were returned to their home cages. After each partner-preference test, the mating chamber and dividers were cleaned with Windex and fresh bedding was added to the floor of the mating chamber. See Fig. 1 bottom.

### Recorded behaviors

For all subjects, trained observers recorded the timing of entries into and exits from each of the side compartments. A subject was considered to have entered a compartment when all four paws passed through one of the holes in the clear Plexiglas dividers. Time spent in each compartment was calculated.

For female subjects, the frequency of solicitation behaviors (i.e., hops and darts, ear wiggling) and the timing and the type of sexual stimulations received (i.e., mounts, intromissions, ejaculations) were recorded. A lordosis response (LR) to each sexual stimulation received was measured. Each LR was rated on a 4-point scale (0–3), and a lordosis quotient (LQ) was calculated as the percent of LRs ≥ 2 [17, 18]. Measures of paced-mating behavior were also calculated. Percentage of exits was calculated as the likelihood that a female subject left the male’s compartment following the receipt of each type of sexual stimulation (i.e., mount, intromission, ejaculation). For example, if a female subject received a second stimulation before leaving, the percentage of exiting the male was 0% for the first stimulation and 100% for the second stimulation for an average of 50%. Contact–return latency was calculated as the time (in seconds) that elapsed between receiving a particular sexual stimulation (e.g., mount, intromission, ejaculation), leaving the male’s compartment, and re-entering the male’s compartment.

For male subjects, the timing and the type of sexual stimulation displayed (i.e., mounts, intromissions, ejaculations) were recorded. Mount frequency, intromission frequency, and ejaculation frequency were also calculated.

### Fertility

Female subjects were monitored for signs of pregnancy (e.g., weight gain) within two weeks of the partner-preference test. Female subjects that showed signs of pregnancy were monitored for parturition every 8–10 h starting 20 days after tests of partner preference. Pups were counted at birth, and then sexed and weaned on PD 23.

### Data analysis

Two-tailed independent *t*-tests were calculated to determine the effect of leuprolide treatment on pubertal onset in female and male subjects. To determine the effect of leuprolide on the first estrous cycle, a two-tailed independent *t*-test was calculated on the number of days after vaginal opening until first estrus (i.e., vaginal opening to first estrus post-vaginal opening) during the treatment period (up until PD 50).

For female subjects, the partner-preference test was analyzed with two repeated measure analysis of variance (ANOVA) tests to examine the effect of leuprolide and sex of the stimulus (i.e., male vs. female) on each of the two dependent measures (i.e., time spent with the stimulus rats, number of visits to the stimulus rats). Tukey’s honestly significant difference (HSD) post hoc tests were used to follow up ANOVAs. Any pairwise comparison that exceeded the honestly significant difference was indicated. Two-tailed independent *t*-tests were calculated to determine the effect of leuprolide on each sexual behavior observed, including frequency of solicitation behaviors (i.e., hops and darts, and ear wiggles), percentage of exits, contact–return latency, LR, and LQ.

For male subjects, two separate 4 (tests) × 2 (treatment) repeated measure ANOVA tests were calculated on time spent with the female stimulus, as well as on time spent with the male stimulus, across the four tests. Visits to the female stimulus and visits to the male were analyzed similarly with two separate 4 (tests) × 2 (treatment) repeated measure ANOVA tests. Development of copulatory behavior was analyzed for each type of sexual stimulation (mount, intromission, ejaculation) across the four tests. Frequency and latency were analyzed separately.

Alpha was set at *p* < 0.05 and effect sizes were estimated using partial eta squared or Cohen’s *d*. A Fisher exact test was used to assess differences between the two treatment groups on the frequency of female subjects becoming pregnant. Significant main effects and interactions are described in detail. Unprotected Tukey’s HSD tests were used for pairwise comparisons. Data are reported as means ± standard error of the mean (SEM).

## Results

### Leuprolide delayed puberty in female and male rats

**Fig. 2:**
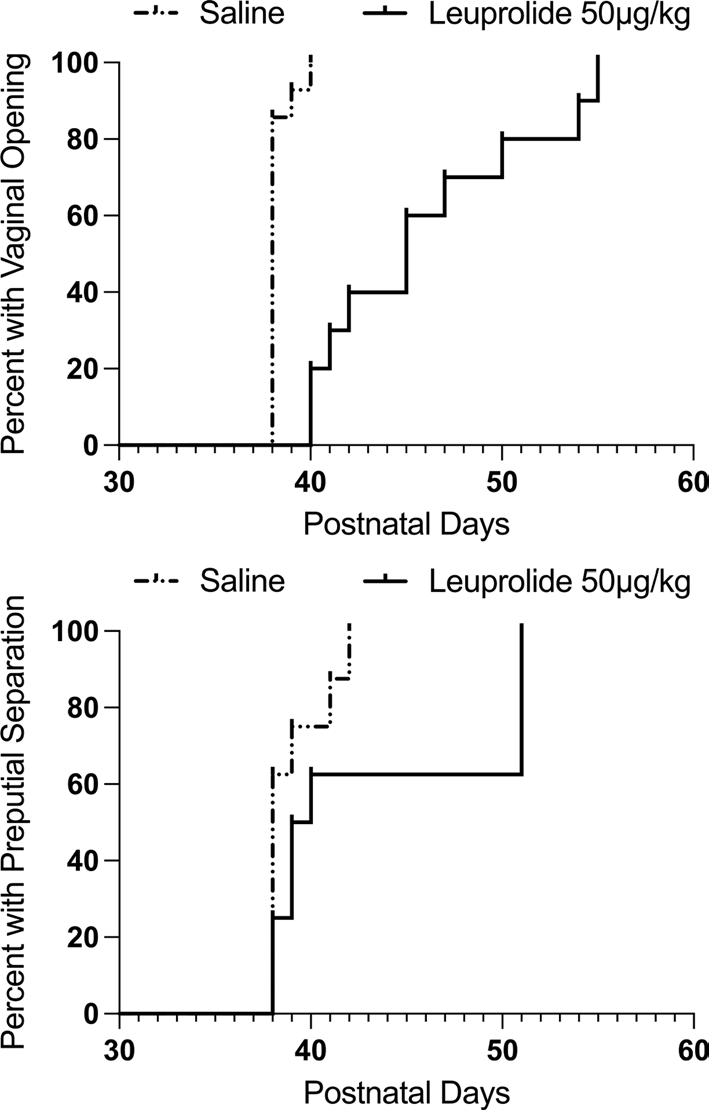
Leuprolide exposure during the periadolescent period delayed pubertal onset. Top: on average, the day of vaginal opening was approximately one week later in leuprolide-treated female rats than saline controls (saline: *n* = 14; leuprolide: *n* = 10). Bottom: day of preputial separation was approximately 6 days later in leuprolide-treated male rats than saline controls (saline: *n* = 8; leuprolide: *n* = 8). Data are presented as the proportion of subjects in each group displaying vaginal opening or preputial separation across time

Treatment with leuprolide (leuprolide *n* = 10; saline *n* = 14) significantly delayed vaginal opening in female subjects [*t*(22) = 5.25, *p* < 0.05, Cohen’s *d* = 2.17]. The average day of vaginal opening for leuprolide-treated female subjects was 45.90 ± 1.75 and the average day of vaginal opening for saline controls was 38.14 ± 0.14 (mean ± SEM). Figure 2 top depicts the cumulative number of subjects displaying vaginal opening across time in each group.

Leuprolide treatment (saline: *n* = 8; leuprolide: *n* = 8) significantly delayed preputial separation in male subjects [*t*(14) = 2.56, p < 0.05, *Cohen’s d* = 1.28]. The average day of preputial separation for leuprolide-treated male subjects was 45.0 ± 2.2 days and the average day of preputial separation for saline controls was 39.0 ± 0.6 days. Figure 2 bottom depicts the cumulative number of subjects displaying preputial separation across time in each group.

### Estrous cyclicity was disrupted during leuprolide treatment

**Fig. 3:**
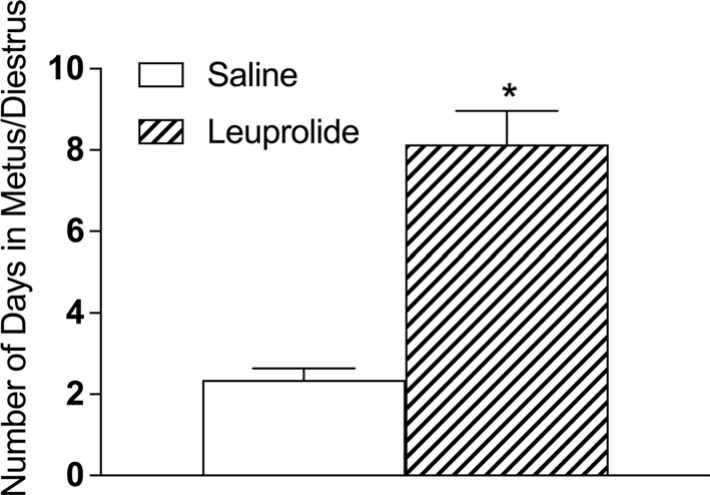
During the period of leuprolide exposure, estrous cyclicity was halted after vaginal opening. Saline controls entered estrus within 4 days of vaginal opening, whereas leuprolide-treated female rats failed to enter estrus after vaginal opening during treatment (saline: *n* = 14; leuprolide: *n* = 7). Data are presented as the mean number of days until first estrus after vaginal opening for each group (± SEM); *n* = number of subjects in each group

Three of the 10 leuprolide-treated female subjects failed to show signs of puberty during the treatment period (i.e., before PD 50). Therefore, only 7 leuprolide-treated female subjects were included in the analysis of days until first estrus after vaginal opening. Treatment with leuprolide significantly disrupted the first estrous cycle after vaginal opening. Female subjects treated with leuprolide failed to enter estrus during the treatment period, whereas all saline controls went into estrus within 4 days of vaginal opening. As such, leuprolide-treated female subjects spent significantly more days in metestrus or diestrus than saline controls during their first estrous cycle after vaginal opening [*t*(19) = 8.20, *p* < 0.05, *Cohen’s d* = 3.79]. See Fig. 3 and Table 1.

**Table 1:**
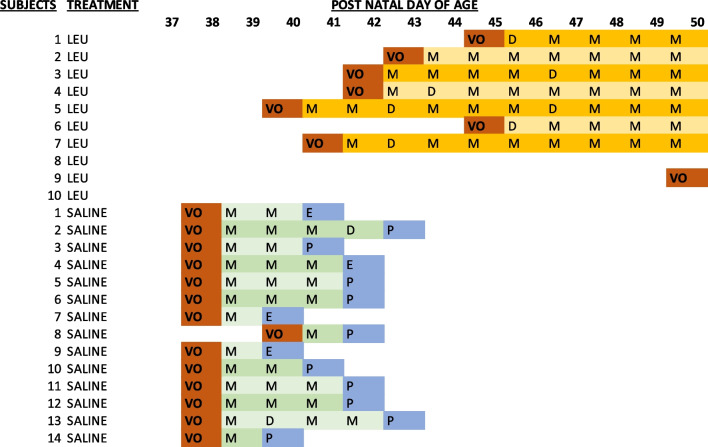
Measurements of vaginal opening and assessment of vaginal cytology daily until the end of the treatment period

*VO* vaginal opening, *P* proestrus, *M* metestrus, *D* diestrus

### Partner-preference test in female rats

Two of the 10 leuprolide-treated female subjects and one of the 14 saline controls failed to go into behavioral estrus within 14 days of treatment termination. Therefore, only 8 leuprolide-treated female subjects and 13 saline controls were included in the analysis of sexual motivation. The female rats treated with leuprolide went into estrus on an average of 7.62 ± 1.3 days after treatment termination. Saline controls went into estrus on an average of 5.20 ± 0.82 days after saline injections ended. This difference was not statistically significant [*t*(19) = 1.66, *p* > 0.05].

### Leuprolide had no effect on sexual motivation or sexual behavior in female rats

#### Time spent with the stimulus rats

**Fig. 4:**
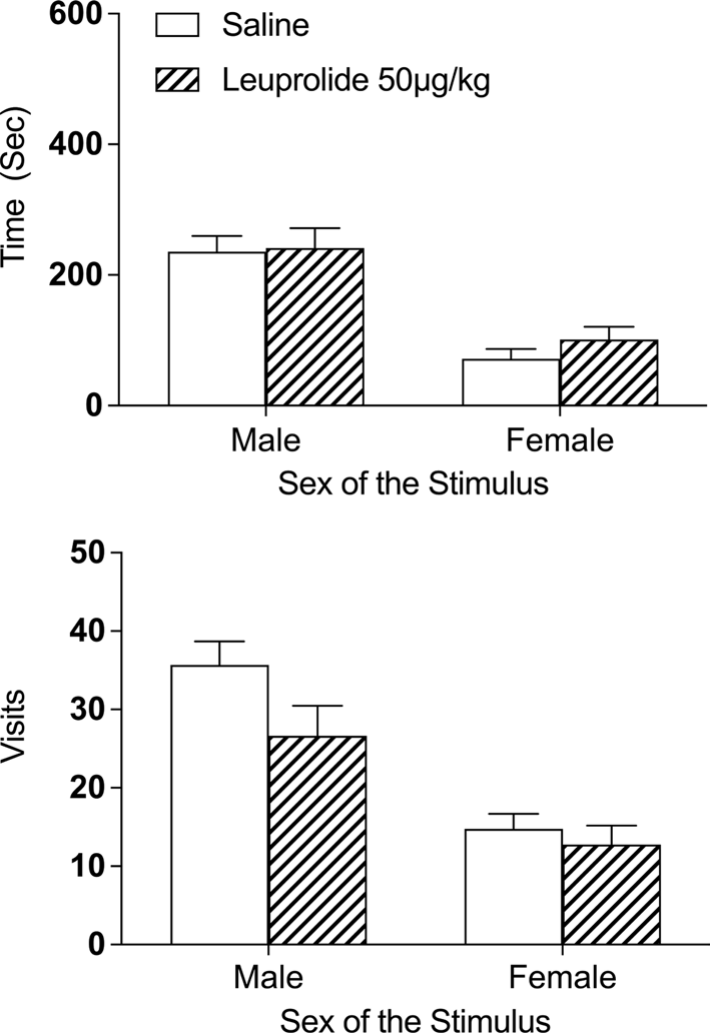
Leuprolide exposure during the periadolescent period had no effect on interest in the male stimulus during the partner-preference test in female rats. Top: independent of leuprolide treatment, all female rats spent more time with the male stimulus than the female stimulus (saline: *n* = 13; leuprolide: *n* = 8). Bottom: similarly, all female rats visited the male stimulus more frequently than the female stimulus, independent of leuprolide treatment. Data are presented as means (± SEM) during the entire 10-min test; *n* = number of subjects in each group

Leuprolide treatment had no effect on sexual motivation during the partner-preference test. See Fig. 4 top. There was a significant main effect of sex of the stimulus on time spent with the stimulus rats [*F*(1,19) = 28.66, *p* < 0.001, $\eta_{p}^{2}$  = 0.60], such that both groups of female subjects spent more time with the male stimulus than the female stimulus, independent of leuprolide treatment. All other main effects and interactions failed to reach statistical significance [*F’s* < 1.2 and *p’s* > 0.05].

#### Visits to stimulus rats

There was a significant main effect of sex of the stimulus rats on visits [*F*(1,19) = 61.90, *p* < 0.001, *η*_*p*_^2^ = 0.76], such that both groups of female subjects visited the male stimulus more frequently than the female stimulus, independent of leuprolide treatment. See Fig. 4 bottom. All other main effects and interactions failed to reach statistical significance [*F’s* < 1.5 and *p’s* > 0.05].

#### Paced-mating behaviors

Finally, leuprolide had no effect on any of the other sexual behaviors recorded during the partner-preference test (e.g., frequency of solicitation behaviors, contact–return latency, percentage of exits, LR, LQ; See Table 2; all *t*’s < 1.6 and *p*’s > 0.05).

**Table 2:** Female mating behaviors calculated during the partner-preference test (means ± SEM)

Treatment	Contact–return latency			Percentage of exits			Receptive behavior		Total solicitations
	Mount	Intromissions	Ejaculations	Mount	Intromissions	Ejaculations	LQ	LR	
Saline	7.6 ± 1.4	12.8 ± 2.0	17.6 ± 3.8	62.5 ± 12.8	92.5 ± 3.3	100.0 ± 0.0	100.0 ± 0.0	2.0 ± 0.0	3.8 ± 1.8
Leuprolide	10.3 ± 1.6	21.2 ± 7.3	27.2 ± 8.2	70.0 ± 10.5	91.6 ± 5.4	100.0 ± 0.0	100.0 ± 0.0	2.0 ± 0.0	9.0 ± 2.3

### Leuprolide had no effect on fertility in female rats

All 13 of the saline controls and all 8 of the leuprolide-treated subjects received an ejaculation during the partner-preference test. As a result of receiving an ejaculation, 4 of the 13 saline controls (31%; producing 39 pups with a mean of 9.75 pups per litter) and 5 of the 8 leuprolide-treated subjects (62.5%; producing a total of 53 pups with a mean of 10.6 pups per litter) became pregnant. Because at least one expected value was less than 5, a Fisher exact test was used and indicated a two-tailed exact significance of *p* = 0.203. Therefore, the difference in pregnancy frequency between the groups (i.e., leuprolide-treated and the saline controls) was not beyond what would be expected by chance.

### Leuprolide had no effect on ovarian and uterine horn weights

On PD 108, approximately 45 days after partner-preference tests, mated subjects were anesthetized with sodium pentobarbital anesthesia (50 mg/kg intraperitoneal (i.p.); Covetrus North America, Chicago, IL, USA) and ovaries and uterine horns were removed and weighed. We analyzed nulliparous females separately. In nulliparous females, there was no effect of leuprolide treatment on raw ovarian weight [*t*(10) = 1.21, *p* > 0.05] (means ± SEM: *n* = 3 leuprolide subjects: 0.96 g ± 0.25 and *n* = 9 saline controls: 1.50 g ± 0.24). There was also no effect of leuprolide treatment on raw uterine weight [*t*(10) = 1.52, *p* > 0.05] (means ± SEM: leuprolide subjects: 2.28 g ± 0.29 and saline controls: 2.88 g ± 0.21). In females who gave birth, there was no effect of leuprolide treatment on raw ovarian weight [*t*(7) < 1.0, *p* > 0.05] (means ± SEM: *n* = 5 leuprolide subjects: 0.67 g ± 0.09 and *n* = 4 saline controls: 0.77 g ± 0.09). Finally, there was no effect of leuprolide treatment on raw uterine weight [*t*(7) < 1.0, *p* > 0.05] (means ± SEM: leuprolide subjects: 1.36 g ± 0.22 and saline controls: 1.39 g ± 0.13).

### Development of partner preference across four tests in male rats

#### Leuprolide delayed the maturation of sexual motivation and copulatory behavior in male rats

##### Time spent with the male stimulus

**Fig. 5:**
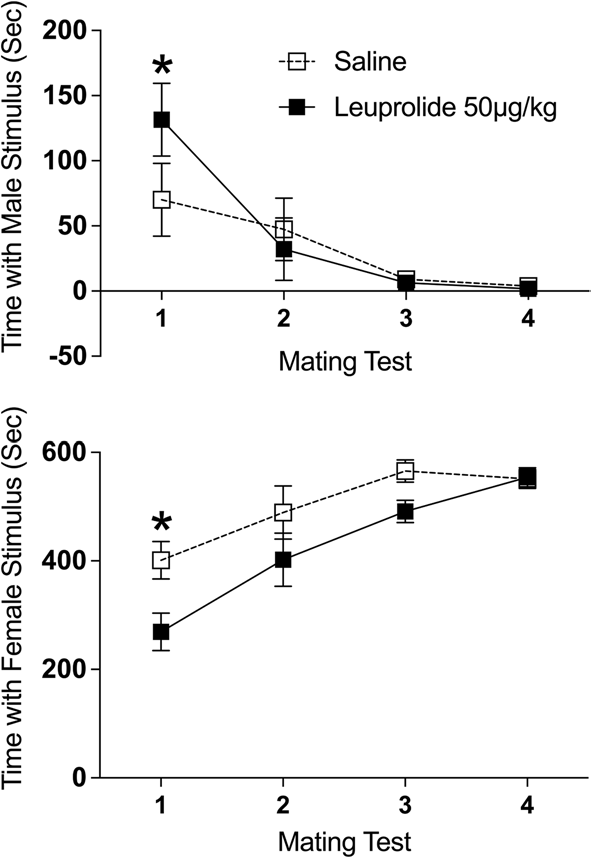
Leuprolide exposure during the periadolescent period increased interest in the male stimulus but decreased interest in the female stimulus during the partner-preference tests in male rats. Top: leuprolide-treated male rats spent more time with the male stimulus than the saline controls (saline: *n* = 8; leuprolide: *n* = 8) on Test 1. Bottom: leuprolide-treated male rats spent less time with the female stimulus than the saline controls collapsed across tests. However, post hoc tests indicate that there was only a significant difference between groups on Test 1. Note: the axis range of the two graphs is not the same. Data are presented as means (± SEM) during the entire 10-min test; *n* = number of subjects in each group. An asterisk (*) indicates significant post hoc difference between groups

There was a significant main effect of repeated tests on time spent with the male stimulus [*F*(3,42) = 11.47, *p* < 0.001, $\eta_{p}^{2}$ = 0.45]. Independent of leuprolide treatment, male subjects spent less time with the male stimulus across the four tests. See Fig. 5 top. Although the interaction between leuprolide treatment across tests was not statistically significant [*F*(3,42) = 1.68, *p* > 0.05], Tukey’s HSD tests indicated that leuprolide-treated male subjects spent significantly more time with the male stimulus than the saline controls on Test 1 (*p* < 0.05) but not on Tests 2–4 (*p’s* > 0.05). See Fig. 5 top.

*Time spent with the female stimulus*: There was a significant main effect of repeated tests on time spent with the female stimulus [*F*(3,42) = 19.37, *p* < 0.001, $\eta_{p}^{2}$ = 0.58]. Independent of leuprolide treatment, male subjects spent more time with the female stimulus across the four tests. See Fig. 5 bottom. There was also a significant main effect of leuprolide treatment on time spent with the female stimulus [*F*(1,14) = 7.87, *p* < 0.05, $\eta_{p}^{2}$ = 0.36]. Leuprolide-treated male subjects spent significantly less time with the female stimulus than saline controls collapsed across the four tests. Although the interaction between leuprolide treatment across tests was not statistically significant [*F*(3,42) = 1.58, *p* > 0.05], Tukey’s HSD post hoc tests indicated that leuprolide-treated male subjects spent less time with the female stimulus than the saline controls on Test 1 (*p* < 0.05) but not on Tests 2–4 (*p’s* > 0.05). See Fig. 5 bottom.

**Fig. 6:**
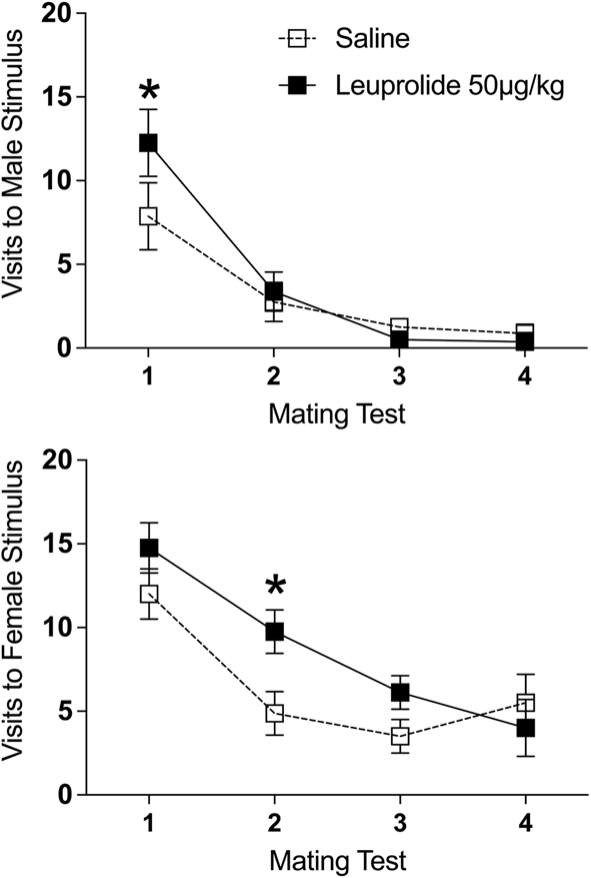
Leuprolide exposure during the periadolescent period affected visits to the stimulus animals during the partner-preference tests in male rats. Top: leuprolide-treated male rats visited the male stimulus more frequently than the saline controls on Test 1 (saline: *n* = 8; leuprolide: *n* = 8). Bottom: leuprolide-treated male rats visited the female stimulus more frequently than the saline controls on Test 2. Data are presented as means (± SEM) during the entire 10-min test; *n* = number of subjects in each group. An asterisk (*) indicates significant post hoc difference between groups. A double asterisk (**) indicates a significant main effect of treatment

*Visits to the male stimulus*: There was a significant main effect of repeated tests on visits to the male stimulus [*F*(3,42) = 19.640, *p* < 0.001, $\eta_{p}^{2}$ = 0.58]. Independent of leuprolide treatment, male subjects visited the male stimulus less frequently across the four tests. See Fig. 6 top. Although the interaction between leuprolide treatment across tests was not statistically significant [*F*(3,42) = 1.41, *p* > 0.05], Tukey’s HSD post hoc tests indicated that leuprolide-treated male subjects visited the male stimulus more frequently than the saline controls on Test 1 (*p* < 0.05) but not on Tests 2–4 (*p’s* > 0.05). See Fig. 6 top.

*Visits to the female stimulus*: There was a significant main effect of repeated tests on visits to the female stimulus [*F*(3,42) = 18.02, *p* < 0.001, $\eta_{p}^{2}$ = 0.56]. Independent of leuprolide treatment, male subjects visited the female stimulus less frequently across the four tests. See Fig. 6 bottom. Although the interaction between leuprolide treatment across tests was not statistically significant [*F*(3,42) = 1.94, *p* > 0.05], Tukey’s HSD post hoc tests indicated that leuprolide-treated male subjects visited the female stimulus more frequently than the saline controls on Test 2 (*p* < 0.05) but not on Tests 1, 3 or 4 (*p’s* > 0.05). See Fig. 6 bottom.

**Fig. 7:**
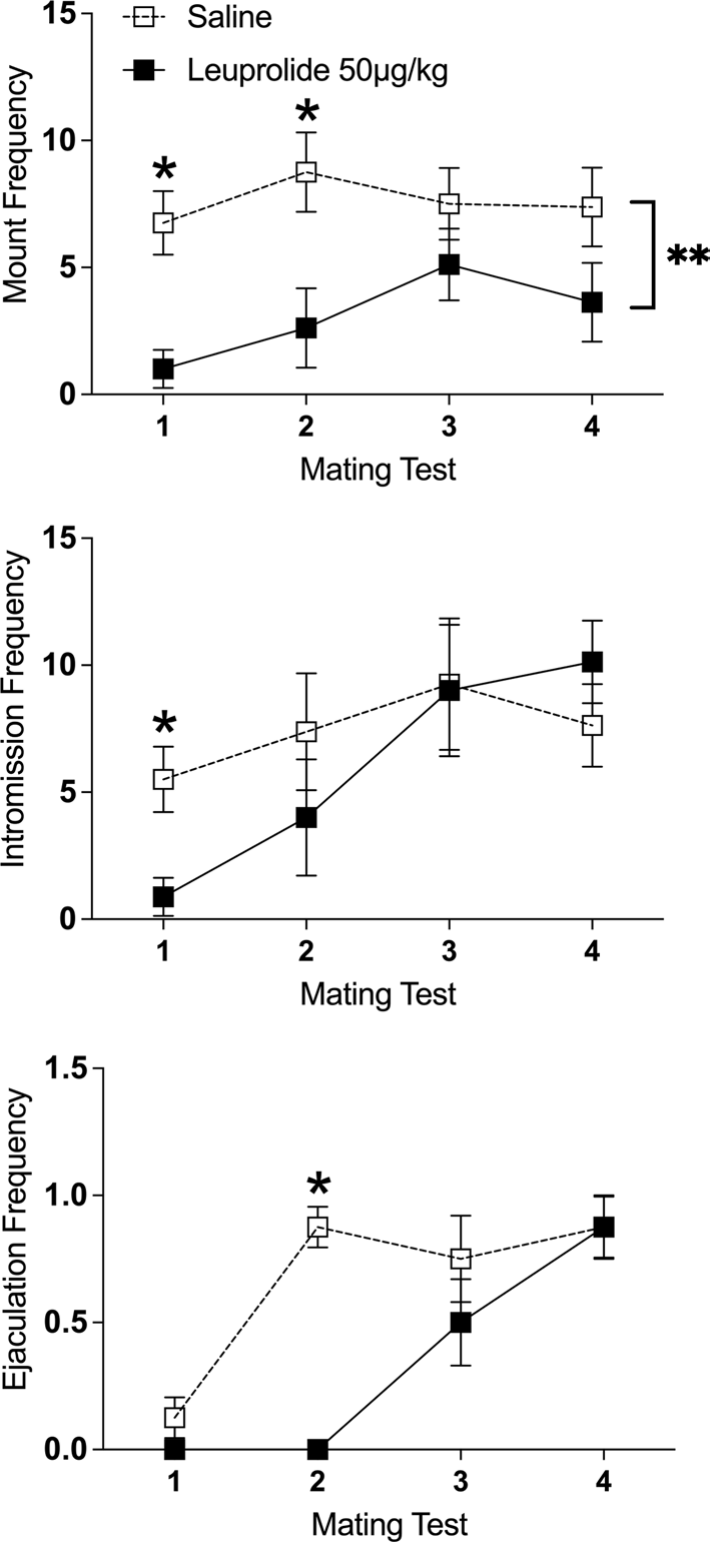
Leuprolide exposure during the periadolescent period delayed the development of copulatory behavior during the partner-preference tests. Top: leuprolide-treated male rats displayed fewer mounts than saline controls (saline: *n* = 8; leuprolide: *n* = 8) collapsed across tests. However, post hoc tests indicate that treatment groups were different on Test 1 and Test 2. Middle: leuprolide-treated male rats displayed fewer intromissions than saline controls on Test 1. Bottom: leuprolide-treated male rats displayed fewer ejaculations than the saline controls on Test 2. Data are presented as mean frequencies (± SEM) during the entire 10-min test; *n* = number of subjects in each group. An asterisk (*) indicates significant post hoc difference between groups. A double asterisk (**) indicates a significant main effect of treatment

*Frequency of copulatory behavior*: For *mounts*, there was a significant main effect of leuprolide treatment on mount frequency, such that leuprolide-treated male subjects displayed fewer mounts than saline controls collapsed across the four tests [*F*(1,14) = 13.50, *p* < 0.01, $\eta_{p}^{2}$ = 0.49]. See Fig. 7 top. Although the interaction between leuprolide treatment across tests was not statistically significant [*F*(3,42) < 1.0, *p* > 0.05], Tukey’s HSD tests indicated that leuprolide-treated male subjects displayed fewer mounts than the saline controls on Test 1 (*p* < 0.05) and on Test 2 (*p* < 0.05) but not on Tests 3 or 4 (*p’s* > 0.05). See Fig. 7 top.

For *intromissions*, there was a significant main effect of repeated tests [*F*(3,42) = 7.21, *p* < 0.001, $\eta_{p}^{2}$ = 0.34], such that the number of intromissions increased across the four tests, independent of leuprolide treatment. See Fig. 7 middle. Although the interaction between leuprolide treatment across tests was not statistically significant [*F*(3,42) = 2.12, *p* > 0.05, $\eta_{p}^{2}$ = 0.13], Tukey’s HSD post hoc tests indicated that leuprolide-treated male subjects displayed significantly fewer intromissions than saline controls on Test 1 (*p* < 0.05) but not on Tests 2–4 (*p’s* > 0.05). See Fig. 7 middle.

For *ejaculations*, there was a significant main effect of repeated tests [*F*(3,42) = 22.10, *p* < 0.001, $\eta_{p}^{2}$ = 0.61], such that the number of ejaculations increased across the four tests. See Fig. 7 bottom. There was also a significant main effect of leuprolide treatment on ejaculation frequency, indicating that leuprolide-treated subjects displayed fewer ejaculations than saline controls collapsed across the four tests [*F*(1,14) = 6.36, *p* < 0.05, $\eta_{p}^{2}$ = 0.31]. Finally, there was a significant interaction between leuprolide treatment across repeated tests [*F*(3,42) = 7.12, *p* < 0.001, $\eta_{p}^{2}$ = 0.34]*.* Tukey’s HSD post hoc tests indicated that leuprolide-treated male subjects displayed significantly fewer ejaculations than saline controls on Test 2 (*p* < 0.05) but not on Tests 1, 3 or 4 (*p’s* > 0.05). See Fig. 7 bottom.

*Latency to display copulatory behavior*: Because only one leuprolide-treated subject (12.5%) displayed mounts during all four tests and only one leuprolide-treated subject (12.5%) displayed intromissions during all four tests, latency to mount or intromit across the four tests could not be analyzed for any effect of leuprolide. Because no leuprolide-treated subject or saline control ejaculated during all four tests, latency to ejaculate across tests could not be analyzed for any effect of leuprolide. Nevertheless, independent sample *t*-tests on each test indicated that on Test 2, leuprolide-treated male subjects took longer to intromit [*t*(8) = 2.42, *p* < 0.05, *Cohen’s d* = 1.67] than saline controls. In addition, leuprolide-treated male subjects took longer to ejaculate than saline controls on Test 3 [*t*(8) = 3.08, *p* > 0.01, *Cohen’s d* = 1.99] and Test 4 [*t*(12) = 3.00, *p* > 0.01, *Cohen’s d* = 1.61]). See Table 3.

**Table 3:** Male copulatory measures observed during the partner-preference test (means in seconds ± SEM)

Treatment	Test	Latency		
		Mount	Intromission	Ejaculation
Saline				
	1	198.8 ± 75.4	269.0 ± 74.1	547.0 ± 0.0
	2	87.5 ± 38.5	95.3 ± 34.6	281.4 ± 73.9
	3	80.7 ± 35.5	59.1 ± 9.0	275.7 ± 27.4
	4	69.7 ± 16.0	71.5 ± 17.5	254.7 ± 30.5
Leuprolide				
	1	117.5 ± 38.5	303.0 ± 197.0	–
	2	223.0 ± 34.5	241.0 ± 42.1*	–
	3	173.5 ± 70.8	164.0 ± 72.3	456.5 ± 60.4*
	4	187.8 ± 53.7	175.9 ± 64.9	438.4 ± 52.9*

Asterisk (*) indicates difference between groups on each test

## Discussion

### Physiological effects of leuprolide

#### Female rats

In the current study, daily leuprolide administration at a dose of 50 µg/kg delayed the onset of puberty by an average of 7 days in female rats. These results are consistent with our previous findings in female rats using the same dose [11]. Nevertheless, most of the female rats treated with leuprolide showed physiological signs of pubertal onset during the treatment period, suggesting that this dose of leuprolide is not able to indefinitely suppress puberty in female rats.

We report here that estrous cyclicity was disrupted during leuprolide treatment when measured immediately after vaginal opening. Specifically, female rats treated with leuprolide failed to enter into estrus or proestrus during the treatment period. Leuprolide-treated female rats stayed in metestrus/diestrus, whereas all saline controls entered estrus within 4 days of vaginal opening. These results indicate that although vaginal opening occurred, leuprolide suppressed the pulsatile release of luteinizing hormone (LH) necessary for estrous cyclicity. This effect of leuprolide on estrous cyclicity is consistent with findings in female mice [10] and rats [19]. For example, two weeks of leuprolide injections suppressed estrous cyclicity in adolescent female mice, such that female mice were persistently in metestrus by the end of the 2 weeks [10]. Similarly, adult female rats that received 21 days of continuous leuprolide infusions went into persistent diestrus within three days of starting treatment [19]. However, neither study measured when estrous cyclicity emerged following treatment termination.

In the present study, we found evidence of vaginal opening but failure to enter estrus during leuprolide treatment. These pubertal landmarks are typically temporarily linked, but that was not the case here. Vaginal opening occurs in response to the first preovulatory increase in ovarian estradiol and its actions on estrogen-sensitive vaginal tissues. Vaginal opening in rats is roughly equivalent to menarche (first menstruation) in primates. Our finding that estrous cyclicity did not immediately begin after vaginal opening suggests that the normal hormonal fluctuations that happen due to follicular development, ovulation, and formation of a corpus luteum were affected by leuprolide suppression of the hypothalamic–pituitary–gonadal axis in rats. It is notable that in humans and non-human primates, menarche can happen months or years before first ovulation (comparable to first estrus in rodents) [20].

Although we do not know the mechanism of the above events, it is likely that hypothalamic neuropeptides interact with one another, with GnRH neurons playing key roles. Kisspeptin expressing neurons in the anteroventral periventricular nucleus (AVPV) project to GnRH neurons, thereby contributing to the initiation of puberty and activation of reproductive circuits [21, 22]. These AVPV kisspeptin neurons are involved in positive feedback of ovarian steroid hormones on the preovulatory GnRH/LH surge [23]. At the same time, kisspeptin neurons in the arcuate nucleus (ARC) that co-express neurokinin B (tachykinin) and prodynorphin—the so-called “KNDy” neurons—regulate steroid negative feedback and pulsatile GnRH secretion, and subsequently LH release [23–25]. We propose that this dichotomy of kisspeptin regulation may be differentially regulated by leuprolide. Consistent with this, tachykinin knockout mice (*Tac1*/*Tac2* KO) showed signs of pubertal onset (i.e., vaginal opening), but failed to cycle into estrus [26], suggesting that tachykinin activation of kisspeptin neurons contributes to estrous cyclicity but is not essential for vaginal opening. Further work studying the kisspeptin and KNDY neurons in the AVPV and ARC, and developmental changes in LH and estradiol, would be beneficial to understanding the mechanisms for disparate actions of leuprolide on vaginal opening and estrous cyclicity.

Nevertheless, despite observing a delay in vaginal opening and a persistent disruption of cyclicity during treatment in the present study, our current study found that within a week of leuprolide treatment termination (PD 51–57), the majority of leuprolide-treated female rats (5 of 8) entered estrus and were sexually receptive. Furthermore, our previous study found that regular estrous cyclicity was observed 2 weeks after leuprolide treatment ended [11]. Together these results indicate that leuprolide may be useful to indefinitely suppress LH, but within days of discontinuing treatment, regular ovarian cyclicity occurs.

Daily leuprolide treatment had no effect on fertility in the female rats, and although the proportion of saline controls that became pregnant was lower than the proportion of leuprolide-treated females that became pregnant, the difference was not statistically significant. One ejaculation may not be enough to ensure pregnancy, but nevertheless, leuprolide did not significantly disrupt fertility under these less-than-ideal circumstances. When tested within the first seven days of treatment cessation (from PD 51–57; leuprolide: 5 of 8; saline controls: 12 of 13), most became pregnant (leuprolide: 3 of 5; saline: 4 of 4). Therefore, full recovery from leuprolide treatment can take place within a week of treatment termination in female rats. These results are consistent with our previous study, which also found no evidence that leuprolide affected fertility within two months of treatment termination [11]. However, in our previous study not all female rats received an ejaculation, so we were not able to thoroughly evaluate fertility [11]. It is unlikely that there are any lasting effects of leuprolide on fertility once treatment ends.

Also consistent with our previous findings [11], we found no long-term effects of leuprolide on ovarian or uterine horn weights, when measured approximately two months after treatment ended. Prior studies suggest that ovarian and uterine horn weights would likely be reduced if measured at the end of leuprolide treatment (i.e., ~ PD 51). For instance, depot injections or daily oral administration of leuprolide reduced ovarian and uterine horn weights at the end of a 35-day treatment period in rats [27]. Daily injections or continuous infusions of leuprolide for 21 days have been shown to reduce uterine horn weights [19]. Our current results show that any effects of leuprolide on these endpoints are not long-lasting.

#### Male rats

In the current study, daily leuprolide administration at a dose of 50 µg/kg delayed the onset of puberty by an average of 6 days in male rats. This delay in preputial separation is about two days longer than what we reported in our previous study [9]. The difference between our two studies is likely a function of administering a higher dose in the present experiment (50 μg/kg here vs. 25 μg/kg previously). Nevertheless, all of the male rats treated with leuprolide showed physiological signs of pubertal onset during the treatment period, suggesting that this dose of leuprolide is not able to indefinitely suppress puberty in male rats.

Although we did not measure serum testosterone at the end of the present experiment, we had previously found that approximately 4 weeks after leuprolide treatment ended, serum testosterone levels increased beyond control levels, probably representing an overshoot as the hypothalamic–pituitary–gonadal axis became activated [9]. Similarly, approximately 3 weeks after serum leuprolide levels neared zero following subcutaneous microcapsule injections, testosterone concentrations attained control levels in male rats [28]. Therefore, within weeks of leuprolide treatment cessation, gonadal hormones return to normal in male rats.

### Behavioral effects of leuprolide

#### Female rats

We again [11] failed to find any effect of daily leuprolide treatment on sexual motivation or on any sexual behavior (e.g., lordosis, paced mating behavior, solicitation behaviors) in female rats. Given that testing took place within two weeks of leuprolide treatment ending, we think it is unlikely that the sex differences we observed are due to differences in the timing of behavioral testing relative to the timing of leuprolide treatment. Because most of the leuprolide-treated female rats (5 of 8) went into behavioral estrus within a week of treatment cessation, we think that gonadal hormones reached functioning levels within a week. It is important to note that because female rats failed to enter estrus during leuprolide treatment, sexual behavior during the leuprolide treatment period would have been non-existent and therefore impossible to study (i.e., no lordosis responses possible). Hence, leuprolide exposure likely disrupts the expression of behavioral estrus during treatment, but these effects do not appear to linger.

#### Male rats

Similar to our initial findings [9], daily leuprolide treatment disrupted the development of male copulatory behavior and the expression of sexual motivation. Specifically, exposure to leuprolide reduced the frequency of copulatory behaviors and the time spent with a female stimulus rat. All types of copulatory behaviors (i.e., mounts, intromissions, ejaculations) were affected at some point during the four tests. Given that leuprolide-treated male rats took longer took ejaculate, the effect of leuprolide on copulatory behavior is likely underestimated. However, despite testing a higher dose in the present study, copulatory behavior in leuprolide-treated males was mostly indistinguishable from copulatory behavior in saline controls by the fourth partner-preference test.

Consistent with Anacker et al. [10], we found that compared to saline controls, leuprolide-treated male rats spent more time with and made more visits to the male stimulus, during the first test. We also observed no aggressive behavior in the leuprolide-treated males when they spent time with the male stimulus. The increase in time spent with and visits to the male stimulus could be related to a decrease in aggression in the leuprolide-treated male rats. For example, Shimshek et al. [29] found that male transgenic mice with deletions of the GluR-B subunit of AMPA receptors on GnRH neurons exhibited significantly less aggressive behavior toward other male mice, whereas controls were more likely to attack other male mice. Essentially, the effects of leuprolide could be a by-product of reduced levels of same-sex aggression, as well as reduced motivation to stay with the female stimulus. Future studies would be necessary to evaluate the impact of leuprolide treatment on aggression.

The disruptive effects of leuprolide on male sexual behavior are likely a consequence of suppressed testicular activity. The eventual development of sexual behavior reflects the time required for gonadal hormones to reach adult levels. As mentioned above, a number of studies indicate that serum testosterone reaches adult levels within weeks of leuprolide termination in rodents. Unfortunately, there is very little known about human adolescents who were treated with GnRH agonists to block pubertal development but decide not to transition or later detransition [30, 31]. We do know that boys treated with leuprolide for precocious puberty reach adult gonadal hormones within 10 months of treatment ending [32, 33]. Furthermore, men prescribed GnRH agonists for prostate cancer also show restoration of testosterone levels within months of treatment cessation [34, 35]. Therefore, it is likely that testosterone levels would reach post-pubertal adult levels within months of discontinued use of GnRH agonists, if an adolescent boy decides not to transition. Based on the present study, the delayed maturation of sexual behavior in male rats parallels the delayed maturation of gonadal hormone secretion. Thus, it is likely that once gonadal hormone levels reach adult levels, young adult men would have no lingering behavioral effects.

Taken together, the different effects of leuprolide on sexual motivation and behavior in male and female rats could reflect not only differences in the sensitivity to leuprolide, but also inherent differences between male and female sexual behavior. Male sexual behavior requires time and experience for full expression [36]. However, lordosis is a reflexive response to physical stimulation during behavioral estrus [18] and paced-mating behavior is observed in sexually naïve female rats [37]. Although female sexual behavior is sensitive to experience, with longer periods of time spent with the male stimulus and quicker return latencies after repeated sexual encounters [38]. These inherent differences in the expression of sexual behavior likely contribute to differences in the effects of leuprolide.

## Summary and conclusions

In summary, chronic daily administration of 50 µg/kg of leuprolide throughout periadolescence delayed pubertal onset in female and male rats. Even though this dose is higher than what we tested previously in male rats, puberty eventually emerged in both sexes, suggesting that this dose of leuprolide was not able to indefinitely suppress preputial separation or vaginal opening. Furthermore, leuprolide halted estrous cyclicity during treatment, preventing behavioral estrus. However, once leuprolide treatment ended female rats: (1) began to cycle, (2) displayed normal sexual behavior during estrus, and (3) successfully got pregnant. In contrast, the delay in pubertal onset also delayed the development of sexual motivation and male copulatory behavior. Nonetheless, by continuing to test weekly for four consecutive weeks, we were able to observe improvement in copulatory behavior and robust sexual motivation in male rats exposed to leuprolide, which is likely a function of normalization of gonadal hormones.

## Perspectives and significance

Based on our study results, male rats seem to be more sensitive to leuprolide than females, taking longer to achieve mature sexual behavior and gonadal hormone levels after treatment ends. This difference is likely a function of both inherent sex differences in the development of sexual behavior, as well as differences in the sensitivity to leuprolide. Puberty eventually emerges during treatment and therefore cannot be indefinitely suppressed by leuprolide. Nevertheless, after treatment termination, both sexes eventually develop adult reproductive physiological and behavioral functioning.

## Data Availability

Data can be made available upon request.

## Abbreviations

PD: Postnatal day
i.p: Intraperitoneal
s.c.: Subcutaneous
GnRH: Gonadotropin releasing hormone
PPS: Preputial separation
VO: Vaginal opening
P: Proestrus
E: Estrus
M: Metestrus
D: Diestrus
LR: Lordosis response
LQ: Lordosis quotient
LH: Luteinizing hormone
ARC: Arcuate nucleus
AVPV: Anteroventral periventricular nucleus
SEM: Standard error of the mean
Tukey’s HSD: Tukey’s honestly significant difference
